# Interleukin-18-primed human umbilical cord-mesenchymal stem cells achieve superior therapeutic efficacy for severe viral pneumonia via enhancing T-cell immunosuppression

**DOI:** 10.1038/s41419-023-05597-3

**Published:** 2023-01-28

**Authors:** Yan Liao, Zeqin Fu, Yinfu Huang, Shiduo Wu, Zhen Wang, Shaotang Ye, Weijie Zeng, Guifang Zeng, Duanduan Li, Yulin Yang, Ke Pei, Jian Yang, Zhiwei Hu, Xiao Liang, Junyuan Hu, Muyun Liu, Juan Jin, Cheguo Cai

**Affiliations:** 1 Shenzhen Beike Biotechnology Co., Ltd, Shenzhen, 518054 China; 2 College of Veterinary Medicine, South China Agricultural University, Guangzhou, 510642 China; 3 National-Local Associated Engineering Laboratory for Personalized Cell Therapy, Shenzhen, 518054 China; 4 Department of Nephrology, the First Affiliated Hospital of Zhejiang Chinese Medical University (Zhejiang Provincial Hospital of Traditional Chinese Medicine), Hangzhou, 310000 China; 5 Key Laboratory of Systems Health Science of Zhejiang Province, School of Life Science, Hangzhou Institute for Advanced Study, University of Chinese Academy of Sciences, Hangzhou, 310024 China

**Keywords:** Viral infection, Mesenchymal stem cells

## Abstract

Coronavirus disease 2019 (COVID-19) treatments are still urgently needed for critically and severely ill patients. Human umbilical cord-mesenchymal stem cells (hUC-MSCs) infusion has therapeutic benefits in COVID-19 patients; however, uncertain therapeutic efficacy has been reported in severe patients. In this study, we selected an appropriate cytokine, IL-18, based on the special cytokine expression profile in severe pneumonia of mice induced by H1N1virus to prime hUC-MSCs in vitro and improve the therapeutic effect of hUC-MSCs in vivo. In vitro, we demonstrated that IL-18-primed hUC-MSCs (IL18-hUCMSC) have higher proliferative ability than non-primed hUC-MSCs (hUCMSCcon). In addition, VCAM-1, MMP-1, TGF-β1, and some chemokines (CCL2 and CXCL12 cytokines) are more highly expressed in IL18-hUCMSCs. We found that IL18-hUCMSC significantly enhanced the immunosuppressive effect on CD3^+^ T-cells. In vivo, we demonstrated that IL18-hUCMSC infusion could reduce the body weight loss caused by a viral infection and significantly improve the survival rate. Of note, IL18-hUCMSC can also significantly attenuate certain clinical symptoms, including reduced activity, ruffled fur, hunched backs, and lung injuries. Pathologically, IL18-hUCMSC transplantation significantly enhanced the inhibition of inflammation, viral load, fibrosis, and cell apoptosis in acute lung injuries. Notably, IL18-hUCMSC treatment has a superior inhibitory effect on T-cell exudation and proinflammatory cytokine secretion in bronchoalveolar lavage fluid (BALF). Altogether, IL-18 is a promising cytokine that can prime hUC-MSCs to improve the efficacy of precision therapy against viral-induced pneumonia, such as COVID-19.

## Introduction

The coronavirus disease 2019 (COVID-19), a pneumonia-like disease caused by the virus severe acute respiratory syndrome coronavirus 2 (SARS-CoV-2), became a pandemic in China in early 2020 [1]. The SARS-CoV-2 infection causes substantial lung damage, ranging from mild respiratory illness to severe acute respiratory syndrome and even death [2]. Current coronavirus vaccines have protected most people from infection, and pulmonary symptoms in patients with mild and moderate COVID-19 can be mitigated with regular supportive therapy and effective antiviral therapy [3]. However, no specific drugs or vaccines are currently available to treat severe COVID-19 patients. The typical characteristics of these patients are an excessive immune response, cytokine storm, upregulation of proinflammatory cytokines and chemokines, acute respiratory distress syndrome (ARDS), respiratory and cardiovascular failure, end-organ damage, and even death [4]. Therefore, there is an urgent need for safe and effective therapeutic methods for mitigating lung injuries in severe COVID-19 patients.

Mesenchymal stem cells (MSCs) are nonhematopoietic cells with immunomodulatory, regenerative, and tri-differentiation properties. There have been reports that MSC infusion reduces pathological changes in the lungs and inhibits the inflammatory response induced by the influenza virus in animal models [5–8] and by the influenza virus or coronavirus in clinical trials [9, 10]. Moreover, in a phase 1 trial, safe and well-tolerated human umbilical cord-derived mesenchymal stem cells (hUC-MSCs) therapy was reported in patients with COVID-19 [4]. In a randomized, double-blind, placebo-controlled phase 2 trial, hUC-MSCs treatment was viewed as a safe and potentially effective therapeutic approach for severe COVID-19 patients with lung damage [3]. However, there are also reports of MSC therapy failing in patients with severe viral pneumonia [11, 12]. One reason is the diversity of inflammatory microenvironments in patients and the heterogeneity of hUC-MSCs from various human sources, which limits therapeutic efficacy.

Some studies have found that the local microenvironment could affect immune-related behaviors and MSC therapeutic efficacy [13, 14]. High inflammatory levels in the microenvironment induce the immunosuppression of MSCs, while low inflammatory levels induce the immune promotion of MSCs [15]. To fully harness MSC immunosuppressive activity, they must be activated or primed in vitro [16–18] or in vivo [19]. Multiple factors have been tested in an attempt to increase MSC immunosuppression and therapeutic efficacy, including the pro-inflammatory cytokines tumor necrosis factor-alpha (TNF-α) [20, 21], interferon-gamma (IFN-γ) [22, 23], interleukin-1 alpha/beta (IL-1α/β) [18, 24], interleukin-17A (IL-17A) [16, 25], interleukin-25 (IL-25) [13], either singly or as a combination of IL-1β + TNF-α and TNF-α + IFN-γ [26–28]. Moreover, transforming growth factor-β1 (TGF-β1) [29], lipopolysaccharide (LPS) [30], metformin [31], and Poly(I:C) [32] were also used for MSC priming. It has a special inflammatory factor expression profile in the microenvironment of different diseases or stages of the same disease. In this study, we must identify the crucial inflammatory factor, which is more highly expressed in severe pneumonia induced by the virus.

Interleukin (IL)‐18 is a proinflammatory cytokine belonging to the IL‐1 family, first identified for its interferon‐γ‐inducing properties [33]. IL‐18‐mediated inflammation has largely been studied in animal models of bacterial, viral, parasitic, and fungal infections [34]. Damage in the later phases of COVID‐19 appears to be driven by a cytokine storm, including interleukin IL‐1 family members and secondary cytokines like IL‐6. IL‐18 could participate in this hyperinflammation, as it was previously found to injure the lung tissue of infected animals by regulating both T helper (Th)1 and Th2 responses [35]. IL‐18 is involved in both innate and acquired immune responses since it is released by macrophages after infections and binds to the IL‐18 receptor (IL‐18Rα and IL‐18Rβ) in the cytomembrane of T and NK cells [36]. After binding to the IL‐18Rα subunit, a heterodimeric complex is formed to propagate the intracellular MyD88 signaling that culminates in proinflammatory gene transcription with the activation of NF‐κB [37]. IL‐18 plasma levels are commonly elevated in viral infections, exceeding 1,000 pg/ml during the acute phase of Epstein–Barr virus (EBV) and in human immunodeficiency virus (HIV) infection, particularly in patients with severe cases [38, 39]. In SARS caused by SARS‐CoV‐1, circulating IL‐18 levels peaked 4–6 days after fever onset and normalized during the convalescent period [40]. Collectively, IL-18 is an important cytokine in the proinflammatory microenvironment of influenza virus-induced severe pneumonia and could influence MSC priming in vitro and in vivo.

In this study, IL-18 first showed higher expression in a mouse model of severe pneumonia, and IL-18 was used for priming hUC-MSCs in vitro. Next, we explored how IL-18 affects the characteristics of hUC-MSCs in vitro and assessed the therapeutic efficacy of IL18-primed-hUC-MSCs on H1N1 virus-induced acute severe lung injury in vivo. Lastly, the TGF-β1-mediated mechanism of the therapeutic effects of IL18-primed hUC-MSCs was clarified.

## Materials and methods

### Isolation and culture of hUC-MSCs

hUC-MSCs were obtained and cultured according to previously described methods [41]. Briefly, the umbilical cord was obtained from a healthy pregnant woman after informed consent was obtained. The umbilical cord was rinsed twice with Dulbecco’s phosphate-buffered saline (D-PBS, Invitrogen), cut longitudinally, and the arteries and veins were removed. The soft gel tissues were dissected into small pieces and individually placed on 100 mm tissue culture dishes with low-glucose Dulbecco’s modified Eagle’s medium (L-DMEM, HyClone) supplemented with 5% (v/v) hPL (UltraGRO^TM^-Advanced, GMP Grade, AventaCell BioMedical), as well as 2 mM l-glutamine and 1% penicillin/streptomycin. After 12 days of culture, the umbilical cord tissue was carefully removed. The plates were washed three times with D-PBS; the plastic adherent cell colonies were trypsinized, and cells (Passage 0, P0) were re-seeded for propagating with the growth medium changing every 72 h. Passage 4 (P4) hUC-MSCs were used in all experiments, and all cells were cultured at 37 °C in a humidified atmosphere of 5% CO_2_.

### Preparation of IL-18-primed hUC-MSCs (IL18-hUCMSC)

hUC-MSCs (P3) were grown in T175 flasks (Corning); when they reached >80% confluence, cells were trypsinized and replated at a density of 5000 cells per cm^2^ in T175. After overnight culture, the IL18-hUCMSC was generated by cells pre-stimulated with 100 ng/ml recombinant human IL-18 (Sigma-Aldrich) for 24 h in the complete medium; hUCMSCcon was the cell control without pre-stimulation. Regardless of IL-18 pretreatment, the IL18-hUCMSC and hUCMSCcon were obtained for the following in vitro and in vivo experiments.

### Flow cytometric analysis

Flow cytometric analyses were performed using a BD™ Aria IIu flow cytometer, and data were analyzed with FlowJo7.5 software (Tree Star). The following anti-human antibodies were used: CD73-PE (TY/23), CD90-FITC (5E10), CD105-APC (266), CD34-PE (563), CD45-FITC (HI30), HLA-DR-PerCP (G46-6), and CD3-APC (UCHT1); and the anti-mouse antibodies were: CD3-FITC (17A2), CD4-PE (GK1.5), and CD8-APC (53-6.7). All these antibodies, along with the corresponding isotype control antibodies, were purchased from BD Pharmingen. 5,6-carboxyfluorescein diacetatesuccinimidyl ester (CFSE; Invitrogen) and 7-AAD (BD Pharmingen) were used to stain proliferative and dead cells.

### Characterization and in vitro differentiation of hUCMSCcon and IL18-hUCMSC

To evaluate the expression changes of MSC surface markers between hUCMSCcon and IL18-hUCMSC, flow cytometry was performed using a BD™ Aria IIu flow cytometer. Antibodies used for cytometric analysis were CD73, CD90, CD105, CD34, CD45, and HLA-DR, as mentioned above.

To evaluate the tri-lineage differentiation potential of hUCMSCcon and IL18-hUCMSC, the osteogenic, adipogenic, and chondrogenic differentiation abilities were analyzed in vitro. Briefly, hUCMSCcon and IL18-hUCMSC were cultured in the relevant differentiation media for 2–3 weeks and analyzed by staining with Alizarin Red, Oil Red O, and toluidine blue staining, as previously described [42].

### hUC-MSCs proliferation assay

hUCMSCcon and IL18-hUCMSC were resuspended in DMEM complete medium (supplemented with 5% (v/v) hPL, as well as 2 mM L-glutamine and 1% penicillin/streptomycin), and seeded to a 12-well plate at 10^4^ cells per well. The cells were trypsinized at each indicated time point over seven days, and the cell numbers were directly counted. The population doubling times (DTs) of hUCMSCcon and IL18-hUCMSC were calculated using the following formula: DT = t × [log 2 / (log Nt-logN0)], where Nt is the number of harvested cells, N0 is the number of seeded cells, and t is the culture time.

### Scratch wound assay

Five straight lines were prepared on the back of 30 mm Petri dishes at 1 cm intervals. Then, a 2 ml cell suspension of hUCMSCcon or IL18-hUCMSC (2.5 × 10^5^ cells/ml) was separately added to two Petri dishes and cultivated for 24 h. Next, a scratch line was made with a 10 μl pipetting spear perpendicular to the five baselines, and detached cells were washed with D-PBS (Invitrogen). Then, 2 ml serum-free medium was added to the Petri dishes.

### Human peripheral blood lymphocyte proliferation assays

Human hUC-MSCs (1 × 10^5^ cells) were plated to a 24-well plate (Corning) and cultured for 24 h they were used for the lymphocyte proliferation assay. Human PBMCs were washed twice with D-PBS and stained with CFSE (5 μmol/l, Invitrogen), which was used to assess T-cell proliferation. The cells were then suspended in Roswell Park Memorial Institute (RPMI)1640 at 1 × 10^6^ cells/ml and distributed to 24 well plates (1 ml/well) in the presence or absence of hUC-MSCs. To induce T-cell proliferation, anti-human CD3 and CD28 antibodies (BD Pharmingen; final concentration, 500 ng/ml) were added to the wells. After four days of coculture, the CD3^+^ T-cells were collected and analyzed by flow cytometry.

### Reverse transcription and real-time qPCR

Total RNA was extracted from hUC-MSCs and mouse lung tissues using the TRIzol reagent (Invitrogen), and 1 μg of RNA was reverse transcribed using a RevertAid First Strand cDNA Synthesis Kit (Thermo Scientific). The resulting cDNA was subjected to real-time PCR with the SYBR Green reagent (Roche) using the human and mouse primers listed in Table 1 and Table 2. The relative mRNA abundances were calculated using the ΔCt method, and the gene expression levels were normalized with respect to those of GAPDH.

**Table 1:** Primers used for the amplification of human transcripts by real-time quantitative PCR.

Genes	Forward sequence (5’ to 3’)	Reverse sequence (5’ to 3’)
GAPDH	GTCTCCTCTGACTTCAACAGCG	ACCACCCTGTTGCTGTAGCCAA
VCAM1	GATTCTGTGCCCACAGTAAGGC	TGGTCACAGAGCCACCTTCTTG
ICAM1	AGCGGCTGACGTGTGCAGTAAT	TCTGAGACCTCTGGCTTCGTCA
MMP1	ATGAAGCAGCCCAGATGTGGAG	TGGTCCACATCTGCTCTTGGCA
MMP2	AGCGAGTGGATGCCGCCTTTAA	CATTCCAGGCATCTGCGATGAG
CCL2 (MCP1)	AGAATCACCAGCAGCAAGTGTCC	TCCTGAACCCACTTCTGCTTGG
CCL5 (RANTES)	CCTGCTGCTTTGCCTACATTGC	ACACACTTGGCGGTTCTTTCGG
CCL7 (MCP3)	ACAGAAGGACCACCAGTAGCCA	GGTGCTTCATAAAGTCCTGGACC
CXCL1 (GRO α)	AGCTTGCCTCAATCCTGCATCC	TCCTTCAGGAACAGCCACCAGT
CXCL2 (GRO β)	GGCAGAAAGCTTGTCTCAACCC	CTCCTTCAGGAACAGCCACCAA
CXCL5	CAGACCACGCAAGGAGTTCATC	TTCCTTCCCGTTCTTCAGGGAG
CXCL8 (IL8)	GAGAGTGATTGAGAGTGGACCAC	CACAACCCTCTGCACCCAGTTT
CXCL12 (SDF1)	CTCAACACTCCAAACTGTGCCC	CTCCAGGTACTCCTGAATCCAC
NGF	ACCCGCAACATTACTGTGGACC	GACCTCGAAGTCCAGATCCTGA
TGF-β1	TACCTGAACCCGTGTTGCTCTC	GTTGCTGAGGTATCGCCAGGAA
IGF1	CTCTTCAGTTCGTGTGTGGAGAC	CAGCCTCCTTAGATCACAGCTC
EGF	TGCGATGCCAAGCAGTCTGTGA	GCATAGCCCAATCTGAGAACCAC
FGF2	AGCGGCTGTACTGCAAAAACGG	CCTTTGATAGACACAACTCCTCTC
HGF	GAGAGTTGGGTTCTTACTGCACG	CTCATCTCCTCTTCCGTGGACA
IDO1	GCCTGATCTCATAGAGTCTGGC	TGCATCCCAGAACTAGACGTGC
PGE2	TCAAGATGTACGTGGTGGCC	CAGAAAGGAGTAGACGAAGCC
TSG6 (TNFAIP6)	TCACCTACGCAGAAGCTAAGGC	TCCAACTCTGCCCTTAGCCATC
PD-L1 (CD274)	TGCCGACTACAAGCGAATTACTG	CTGCTTGTCCAGATGACTTCGG
HLA-G	GAAGAGGAGACACGGAACACCA	TCGCAGCCAATCATCCACTGGA
PTGES-2	CCTCTATGAGGCTGCTGACAAG	ATCACACGCAGCACGCCATACA

**Table 2:** Primers used for the amplification of mouse transcripts by real-time quantitative PCR.

Genes	Forward sequence (5’ to 3’)	Reverse sequence (5’ to 3’)
GAPDH	CATCACTGCCACCCAGAAGACTG	ATGCCAGTGAGCTTCCCGTTCAG
IFN-γ	CAGCAACAGCAAGGCGAAAAAGG	TTTCCGCTTCCTGAGGCTGGAT
TNF-α	GGTGCCTATGTCTCAGCCTCTT	GCCATAGAACTGATGAGAGGGAG
IL-1β	TGGACCTTCCAGGATGAGGACA	GTTCATCTCGGAGCCTGTAGTG
IL-6	TACCACTTCACAAGTCGGAGGC	CTGCAAGTGCATCATCGTTGTTC
IL-10	CGGGAAGACAATAACTGCACCC	CGGTTAGCAGTATGTTGTCCAGC
IL-18	GACAGCCTGTGTTCGAGGATATG	TGTTCTTACAGGAGAGGGTAGAC
Viral matrix protein 1 (M1)	GACCRATCCTGTCACCTCTGAC	GGGCATTYTGGACAAAKCGTCTACG

### Viruses and animals

The type of influenza virus we used in our study is the mouse-adapted Influenza A/Puerto Rico/8/34 (H1N1; abbreviated as PR8) and was propagated in 10-day-old SPF chicken embryos at 37 °C for 48 h. Aliquots of collected allantoic fluids were stored at −80 °C in the lab of the College of Veterinary Medicine, South China Agricultural University. The allantoic fluid was collected and titrated to determine the 50% tissue culture infection dose (TCID_50_) in Madin-Darby canine kidney (MDCK) cells and 50% egg infection dose (EID_50_) in chicken embryos. All experiments involving the live virus (PR8) were performed in an approved biosafety level 2 (BSL-2) laboratory.

Eight-week-old specific pathogen-free (SPF) grade female BALB/c mice (body weight: 18–20 g) were purchased from the Animal Center at the Medical Laboratory of Guangdong Province, China. All mice were maintained in a specific pathogen-free facility, and all animal procedures and protocols were reviewed and approved by the animal experimental ethics committee of the South China Agricultural University.

### Infection, monitoring, and sampling of mice

Seventy BALB/c mice were intraperitoneally injected with 0.2 ml 3% (v/v) chloral hydrate. Several minutes later, the 50 μl saline or PR8 (EID_50_) was administered through a nasal inhalation (0 days-post-infection, 0 dpi). BALB/c mice were randomly allocated to four experimental groups: Mock group (10 mice), Model + Saline group (20 mice), Model + hUCMSCcon group (20 mice), and Model + IL18-hUCMSC group (20 mice). After infection with the PR8 virus, the mice in different groups were observed daily, and changes in the clinical symptoms, body weight, and survival were recorded for up to 14 days. Mice that lost more than 20% of their body weight were considered to have reached the experimental endpoint and were euthanized. Model mice were injected intravenously with 100 μl saline without or with hUC-MSCs (hUCMSCcon or IL18-hUCMSC, 1.0 × 10^6^ cells/per mouse) at 3 dpi. At 7 dpi, five mice from each group were sacrificed, and their blood was obtained by excising the eyeballs. Blood samples were then centrifuged at 1000 g for 20 min at 4 °C, and supernatants were collected as serum. Bronchoalveolar lavage fluid (BALF) from both lungs was obtained by three consecutive installations and aspirations of 500 μl sterile D-PBS; aliquots were spun at 800 g 4 °C for 5 min; supernatant from the first lavage was removed and stored at −80 °C for further analysis. The lung tissues in each group were collected and homogenized in 1 ml of sterile D-PBS. At 7 or 14 dpi, newborn rats were sacrificed, and the whole lung tissue was harvested for histology (hematoxylin-eosin, H&E, and Masson’s trichrome staining), and total lung mRNA analysis.

### Lung injury conditions and lung index

The whole lung tissue of the mice was collected at 14 dpi, and the degree of lung injury visible to the naked eye was dark red due to edema. The area ratio of lung injury to the total lung tissue was estimated by at least three different individuals, from which the average was calculated. Finally, the lung injury area of five mice in each group was counted. The wet weight of the lung tissue was weighed. Lung index = lung wet weight/body mass.

### Histopathology

At 7 and 14 dpi, lung tissues were fixed in 4% paraformaldehyde and then dehydrated, embedded in paraffin, and cut into 5 μm-thick sections. The sections were stained with hematoxylin and eosin (H&E) and Masson’s Trichrome using a Leica DM500B microscope (Leica, Germany).

### Quantification of cytokines

Mouse BALF, serum, and lung tissue were harvested to count cytokines. The lung tissue was homogenized in 1 ml of D-PBS containing protease inhibitor cocktail (Roche). The homogenate was centrifuged at 16,000 g for 20 min at 4 °C before the supernatant was harvested. The amounts of the following cytokines were analyzed: IFN-γ, TNF-α, IL-1β, and IL-6. All cytokines were analyzed using a commercial ELISA kit (R&D Systems).

### The blockage of TGF-β1 function

Fresolimumab (GC1008) (MedChemExpress) and TGF beta-1,2,3 monoclonal antibody (eBioscience) were used to block or neutralize the activity of IL18-hUCMSC-derived TGF-β1 in human peripheral blood lymphocyte proliferation assays, and anti-apoptosis experiments of human and mouse lung cells. Human MRC-5 cell line (CL-0161) and primary mouse type II alveolar epithelial cell (CP-M003) were purchased from Procell company (Wuhan, China). The concentration of GC1008 and monoclonal antibody were 30 μg/ml and 10 μg/ml in vitro and in vivo, respectively.

### Statistical analysis

All results represent at least three independent experiments and are expressed as mean ± SEM. All statistical comparisons were made using a two-tailed Student’s *t*-test (between two groups) or one-way ANOVA (for multi-group comparisons). *P* < 0.05 was considered significant. Analysis and graphing were performed using the Prism software (v 5.01, GraphPad).

## Results

### Acute severe lung injury caused by H1N1 virus infection leads to elevated IL-18 expression

**Fig. 1:**
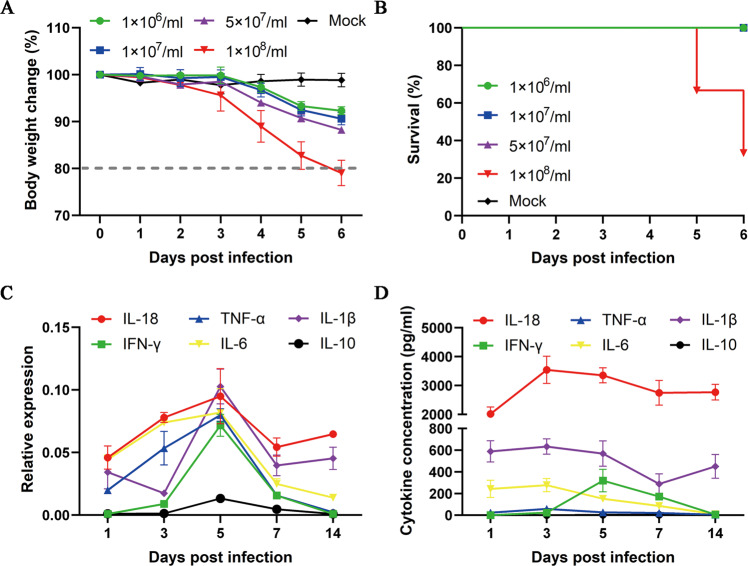
IL-18 is highly expressed in the H1N1 virus-induced mouse model of severe pneumonia. **A** The body weight change in mice withpneumonia induced by four different viral doses, the EID_50_ was 1 × 10^6^/ml, 1 × 10^7^/ml, 5 × 10^7^/ml, and 1 × 10^8^/ml, respectively. Only 1 × 10^8^/ml viral doses could induce severe pneumonia, showing body weight loss of more than 20% at day 6 post-infection; *n* = 5 per group in each time point. **B** The survival rate of different viral dose groups, and the mice death only occurred in the highest dose group; *n* = 5 per group at each time point. **C** The mRNA expression of IL-18 and five important cytokines (TNF-α, IFN-γ, IL-1β, IL-6, and IL-10) in lung tissues were analyzed by qPCR from day 1 to day 14 after H1N1 viral infection; *n* = 3 per group in each time point. **D** The protein concentration of different cytokines in BALF was analyzed using an ELISA kit; *n* = 3 per group at each time point. Data are shown as mean ± SEM; *p < 0.05, **p < 0.01, ***p < 0.001.

To evaluate the therapeutic effect of primed hUC-MSCs on severe lung injury, it is necessary to induce a severe mouse model with a weight loss of more than 20% (reaching the endpoint of the experiment). Four viral infection doses (EID_50_: 1 × 10^6^/ml, 1 × 10^7^/ml, 5 × 10^7^/ml, and 1 × 10^8^/ml) were used in H1N1 (PR8) virus-induced acute lung injuries in vivo. The results demonstrated that the body weight of mice gradually decreased after viral infections, while only the highest infection dose (EID50: 1 × 10^8^/ml) could reduce the body weight to 80% lower than their original level 6 days post-infection (6 dpi) (Fig. 1A), and the survival rate at 5 dpi and 6 dpi decreased to 66.67% and 33.33%, respectively (Fig. 1B). As such, a severe disease mouse model was successfully established.

Various diseases induce unique cytokine expression profiles. In this study, we investigated changes in several important cytokines in a mouse model of severe lung injuries. We found that five key genes (TNF-α, IFN-γ, IL-1β, IL-6, and IL-10) first increased and then decreased, peaking at 5 dpi (Fig. 1C). This phenomenon is consistent with typical inflammatory responses to viral infections.

Interestingly, we confirmed that the gene expression of proinflammatory factor IL-18 in the injured lung was higher than that of the other five cytokines, and its changes were similar to that of these genes (Fig. 1C). In addition, the IL-18 protein concentration in bronchoalveolar lavage fluid (BALF) was higher (approximately 2000–3500 pg/ml) than in the others, which were lower than 650 pg/ml (Fig. 1D). Together, the expression of the proinflammatory factor IL-18 is elevated in the severe H1N1 virus-induced acute lung injury model, suggesting that it could stimulate transplanted hUC-MSCs in vivo.

### IL-18-primed hUC-MSCs exhibit robust immunosuppressive ability

**Fig. 2:**
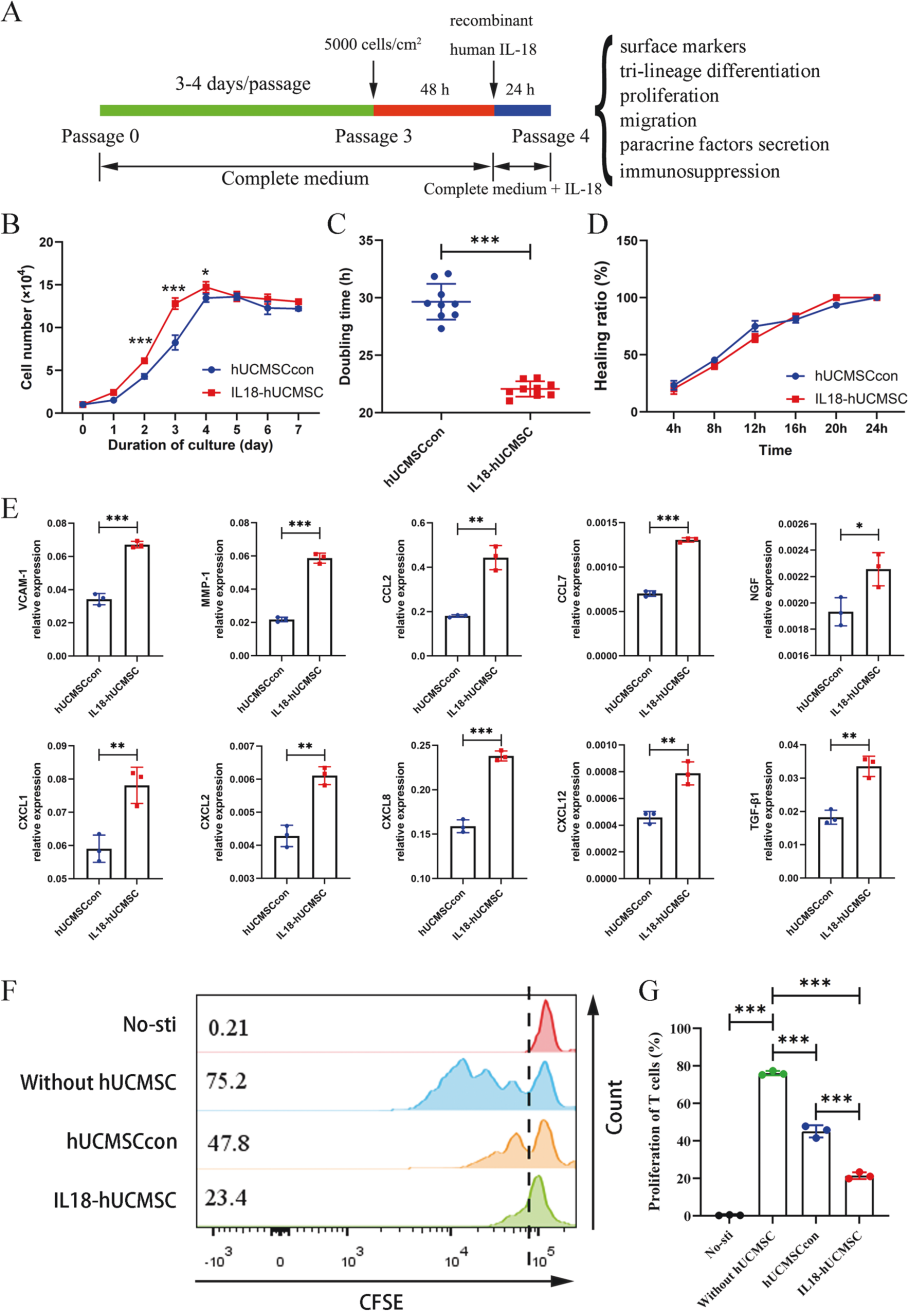
The different properties of IL-18-primed hUC-MSCs were evaluated, and IL18-hUCMSC showed enhanced immunosuppressive ability. **A** Schematic of protocols used to represent the culture and propagation of hUC-MSCs from P 0 to P 3, IL-18 priming time at P 3, and characteristic verification at P 4, including surface markers, tri-lineage differentiation potential, proliferation and migration ability, paracrine factors secretion, and immunosuppression. **B** Growth curves of hUCMSCcon and IL18-hUC-MSC were assessed by direct counting for 7 days. Three replicates were performed at each time point. **C** The doubling time (DT) of hUCMSCcon and IL18-hUC-MSC were analyzed by a formula: DT = t × [log 2 / (log N_t_ – logN_0_)], where N_t_ is the number of harvested cells, N_0_ is the number of seeded cells and t is the culture time. **D** The analysis of healing ratio (%) in scratch wound assay at 4 h, 8 h, 12 h, 16 h, 20 h, and 24 h after scratching. **E** The mRNA expressions of paracrine-related genes (VCAM-1, MMP-1, CCL family, CXCL family, NGF, and TGF-β1) in hUCMSCcon and IL18-hUCMSC were analyzed by qPCR. **F**, **G** The proliferation level of human CD3^+^ T-cells was analyzed by flow cytometry; the change of CFSE fluorescence intensity indicates the growth ratio (**F**); the immunosuppression ratio was analyzed statistically (**G**). Data are shown as mean ± SEM. *n* = 3–9. *p < 0.05, **p < 0.01, ***p < 0.001.

We first investigated the expression of the IL-18 receptor (IL-18R) in hUC-MSCs to ensure activation of the downstream signaling pathway of IL-18-IL-18R. RT-qPCR analysis indicated that IL-18R expression is ~1/500th that of GAPDH (Supplementary Fig. 1A). To better understand IL-18R expression levels, we compared the expression of several common factor receptors, including IFN-γ receptors (IFNGR1 and IFNGR2), TNF receptors (TNFR1 and TNFR2), TGF-β receptors (TGFbR1 and TGFbR2), IL-1 receptors (IL1R1 and IL1R2), and the IL-17 receptor (IL17RA). We found that the mRNA expression of IL-18R was higher than that of IL1R2 and lower than that of the other eight receptors (Supplementary Fig. 1B). These results indicate that hUC-MSCs express IL-18R at relatively lower levels. We then investigated the characteristics and functions of IL-18-primed hUC-MSCs, as illustrated in the scheme (Fig. 2A). In detail, hUC-MSCs were first cultured in a complete medium from passage 0 (P 0) to P 3, with passaging every 3–4 days. P 3 hUC-MSCs were re-seeded in a culture dish in a complete medium by the 5000 cells/cm^2^. Recombinant human IL-18 protein was added into a fresh medium after 48 h of culture, and the P 4 IL-18-primed hUC-MSCs (IL18-hUCMSC) or control hUC-MSCs (hUCMSCcon) were obtained with or without 24 h of priming. Then, the surface markers, tri-lineage differentiation potential, proliferation ability, migration ability, paracrine secretion, and immunosuppression ability were analyzed in the following experiments. First, we performed immunophenotyping of IL18-hUCMSC and hUCMSCcon. At P4, more than 95% of these two hUCMSCs were positive for typical mesenchymal cell surface markers (CD73, CD90, and CD105), while hematopoietic cell markers (CD34 and CD45) and HLA-DR were almost completely absent (Supplementary Fig. 2A). We also assessed the ability of IL18-hUCMSC and hUCMSCcon to differentiate into osteocytes, adipocytes, and chondrocytes on day 21 of culture in the conditioned medium. The results indicated that IL18-hUCMSC and hUCMSCcon had similar tri-differentiation abilities (Supplementary Fig. 2B). Second, the results of cell proliferation showed that IL18-hUCMSC expanded faster than hUCMSCcon during the seven-day culture, especially, on days 2 and 3 (Fig. 2B). The population doubling time (DT) was significantly lower for IL18-hUCMSC compared with hUCMSCcon (22.06 ± 0.63 h versus 29.65 ± 1.47 h, Fig. 2C). We found no significant difference in cell migration between IL18-hUCMSC and hUCMSCcon in a scratch wound assay, with a similar healing ratio from 4 h to 24 h (Fig. 2D and Supplementary Fig. 3A). Third, the qPCR analysis demonstrated that IL-18 priming could increase the mRNA expression of vascular cell adhesion molecule-1 (VCAM-1) and matrix metalloproteinase-1 (MMP-1), but not intercellular cell adhesion molecule-1 (ICAM-1) and MMP-2 in the IL18-hUCMSC group, compared with that of the hUCMSCcon group (Fig. 2E, and Supplementary Fig. 3B). Adhesion and matrix degradation are two prerequisites for MSCs to move into injured tissues. Compared to the hUCMSCcon group, many chemokines have increased expression in the IL18-hUCMSC group, including CCL2, CCL7, CXCL1, CXCL2, CXCL8, and CXCL12 (while CCL5 and CXCL5 have no obvious change) (Fig. 2E, and Supplementary Fig. 3B), suggesting that IL18-hUCMSC can recruit a variety of immune cells. Transforming growth factor-beta 1 (TGF-β1), an immunomodulatory factor [43], significantly increased after IL-18 priming in the IL18-hUCMSC group (Fig. 2E), but other IDO, PGE-2, TSG-6, and PD-L1 expressions did not obviously increase compared to hUCMSCcon (Supplementary fig. 3C). In addition, many growth factors were analyzed by qPCR. The expression of nerve growth factor (NGF) in IL18-hUCMSC exceeded that of hUCMSCcon, but many other IGF-1, EGF, FGF-2, and HGF did not increase after IL-18 priming (Fig. 2E, and Supplementary Fig. 3D). According to qPCR data, the most important immunosuppressive capacity of hUC-MSCs was evaluated by an in vitro coculture experiment.

The flow cytometric data in Fig. 2F demonstrates that the proliferation percentage of T-cells not cocultured with hUC-MSCs was 76.10 ± 0.94%. After four days of coculture, hUCMSCcon could significantly suppress the proliferation of T-cells, from 76.10 ± 0.94% to 45.03 ± 2.63%. Importantly, compared with hUCMSCcon, IL18-hUCMSC significantly reduced the inhibition of T-cells (21.43 ± 1.46% versus 45.03 ± 2.63%) (Fig. 2G). IFN-γ was usually used as an effective factor to prime MSCs in vitro for enhancing immunosuppressive ability [17, 22, 23], the immunosuppression of IFN-γ-primed hUC-MSCs and IL18-primed hUC-MSCs was further compared in vitro (Supplementary Fig. 4A). We found that IFN-γ-hUCMSC show stronger immunosuppressive ability than that of IL18-hUCMSC (8.85 ± 2.94% versus 18.50 ± 1.63%) (Supplementary fig. 4B). Further, several immunomodulatory factors were also analyzed by qPCR between IFN-γ-hUCMSC and IL18-hUCMSC. IDO, PD-L1, and HLA-G were expressed very higher in IFN-γ-hUCMSC group than that in IL18-hUCMSC group; reversely, PTGES-2 and TGF-β1 significantly increased after IL-18 priming in the IL18-hUCMSC group than IFN-γ-hUCMSC group, especially TGF-β1 had a higher expression in IL18-hUCMSC group (Supplementary fig. 4C). Together, the induction of hUC-MSCs by IL-18 in vitro promotes MSC proliferation, secreting some adhesion/matrix degradation/chemokine/growth paracrine factors and enhancing the immunosuppressive ability of T-cells; and TGF-β1 should be a potential immunosuppressive factor for IL18-hUCMSC.

### IL18-hUCMSC enhances therapeutic effects by attenuating acute lung injuries in PR8-infected mice

**Fig. 3:**
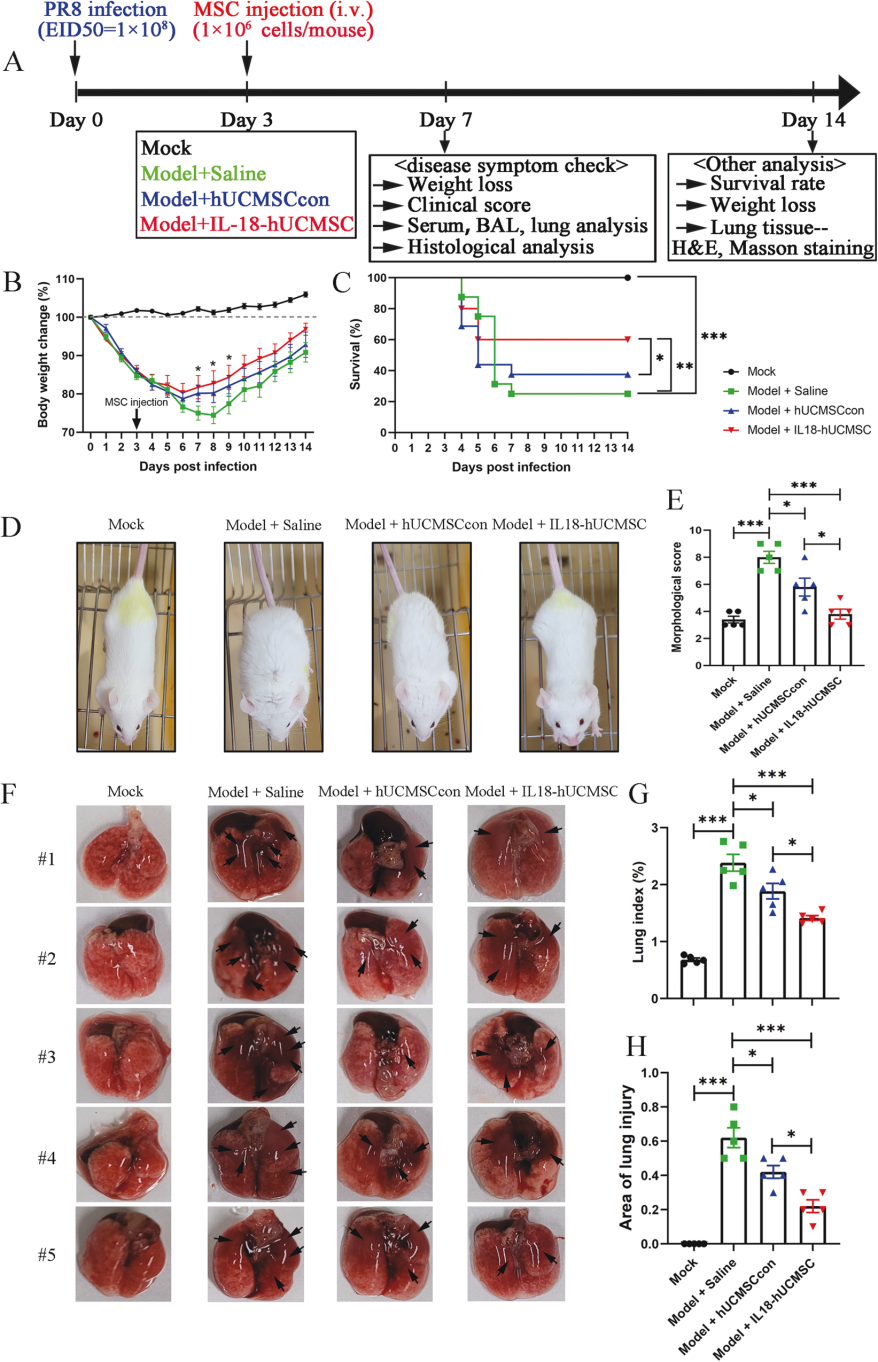
Design scheme of in vivo experiment and IL18-hUCMSC enhanced therapeutic efficacy in the body weight, survival rate, clinical manifestations, and lung injury of BALB/c mice following PR8 infection. **A** Schematic of protocols used for the model establishment, MSC injection, and index analysis. Briefly, the PR8 virus infection dose is 1 × 10^8^ (EID_50_) and BALB/c mice (8 weeks) were intranasally infected PR8 at 0 dpi, hUC-MSCs injection dose is 1 × 10^6^ cells/mouse and was injected intravenously (i.v.) at 3 dpi; Weight change, clinical score, serum, BALF, and lung tissue were analyzed at 7 dpi; survival rate, weight change and Histopathological examination were analyzed at 14 dpi. **B**, **C** From 0 dpi to 14 dpi, the body weight (**B**) and survival rate (**C**) in each point were recorded among saline and hUC-MSCs (hUCMSCcon or IL18-hUCMSC) treatment group. *n* = 10–20. **D**, **E** Clinical symptoms of BALB/c mice after PR8 infection at 7 dpi in different groups were recorded (**D**), and the morphological score was calculated by analyzing reduced activity (0–3 score), ruffled fur (0–3 score), hunched back (0–3 score) and weight loss (0–3 score), with a total of 12 scores (**E**). **F**–**H** Mouse lungs were examined for changes in morphology (**F**), lung index (**G**), and lung injury areas (**H**); Lung index = lung wet weight/body mass; area ratio of lung injury to the total lung tissue was estimated, and the lung injury area of five mice in each group was counted. The arrows in F showed the major changes of the lungs in different groups. Data are shown as mean ± SEM. *n* = 5–10 in each group. *p < 0.05, **p < 0.01, ***p < 0.001.

The schematic of protocols used for establishing a severe lung injury model at day 0, included hUC-MSCs injection (i.v.) at 3 dpi, and analysis of weight loss, survival rate, serum, BALF, and lung tissue at 7 or 14 dpi (Fig. 3A). The body weight of model mice significantly reduced after PR8 infection from 0 to 8 dpi. hUCMSCcon transplantation could increase the body weight from 6 dpi compared with the saline treatment group, but there was no significant difference between these two groups. Importantly, body weight increased in the IL18-hUCMSC group from 5 dpi, and there are significant differences at 7 dpi, 8 dpi, and 9 dpi, compared with the saline treatment group (Fig. 3B). The survival rates significantly decreased in the Model + Saline group compared with the Mock group and Model + IL18-hUCMSC group (25.0% versus 100.0%, and 60.0%, respectively; Fig. 3C). Importantly, model mice with IL18-hUCMSC treatment had higher survival rates than those with hUCMSCcon treatment (60.0% versus 37.5%; Fig. 3C). There was no change in the general appearance of the Mock group mice. In the Model + Saline group, flu-like symptoms began to appear at 4 dpi, such as reduced activity, ruffled fur, hunched back, and weight loss. The symptoms of the Model + hUCMSCcon group were slightly better than those of the Model + Saline group, while IL18-hUCMSC treatment could restore milder clinical symptoms than the Model + hUCMSCcon group (Fig. 3D); the morphological scores in these four groups also had lower scores in the IL18-hUCMSC treatment group, similar with the Mock group (Fig. 3E). This demonstrated that IL18-hUCMSC had enhanced therapeutic effects after assessing clinical symptoms. The results of general lung tissue analysis showed that in PR8-infected mice, the lungs exhibited different degrees of damage, and the color of the injured parts changed from pink to dark red with the presence of edema. The extent of the lung injury in the Model + Saline group was significantly more severe than in the Model + hUCMSCcon group and the Model + IL18-hUCMSC group; the lung color was darker, and the lesion area was larger. Interestingly, the degree of lung injury in the Model + IL18-hUCMSC group was significantly less severe than in the Model + hUCMSCcon group (Fig. 3F). The results of the lung index showed that the lung index of the Model + Saline group significantly increased compared with the Mock group, from 0.682 ± 0.059% to 2.384 ± 0.297%. hUCMSCcon and IL18-hUCMSC treatment reduced the lung index, from 2.384 ± 0.297% to 1.885 ± 0.273% or 1.413 ± 0.086%, respectively (Fig. 3G). The area of lung injury in different groups displayed a similar change trend regarding the lung index; the area was 0.620 ± 0.117, 0.420 ± 0.075, and 0.220 ± 0.075 in the Model + Saline group, Model + hUCMSCcon group, and Model + IL18-hUCMSC group, respectively (Fig. 3H). Figure 3F–H demonstrates that IL-18 priming on hUC-MSCs could significantly decrease lung damage and promote lung repair. Altogether, IL18-hUCMSC showed enhanced therapeutic efficacy in PR8-infected mice.

### IL18-hUCMSC attenuated acute lung injuries by reducing inflammation, fibrosis, and cell apoptosis

**Fig. 4:**
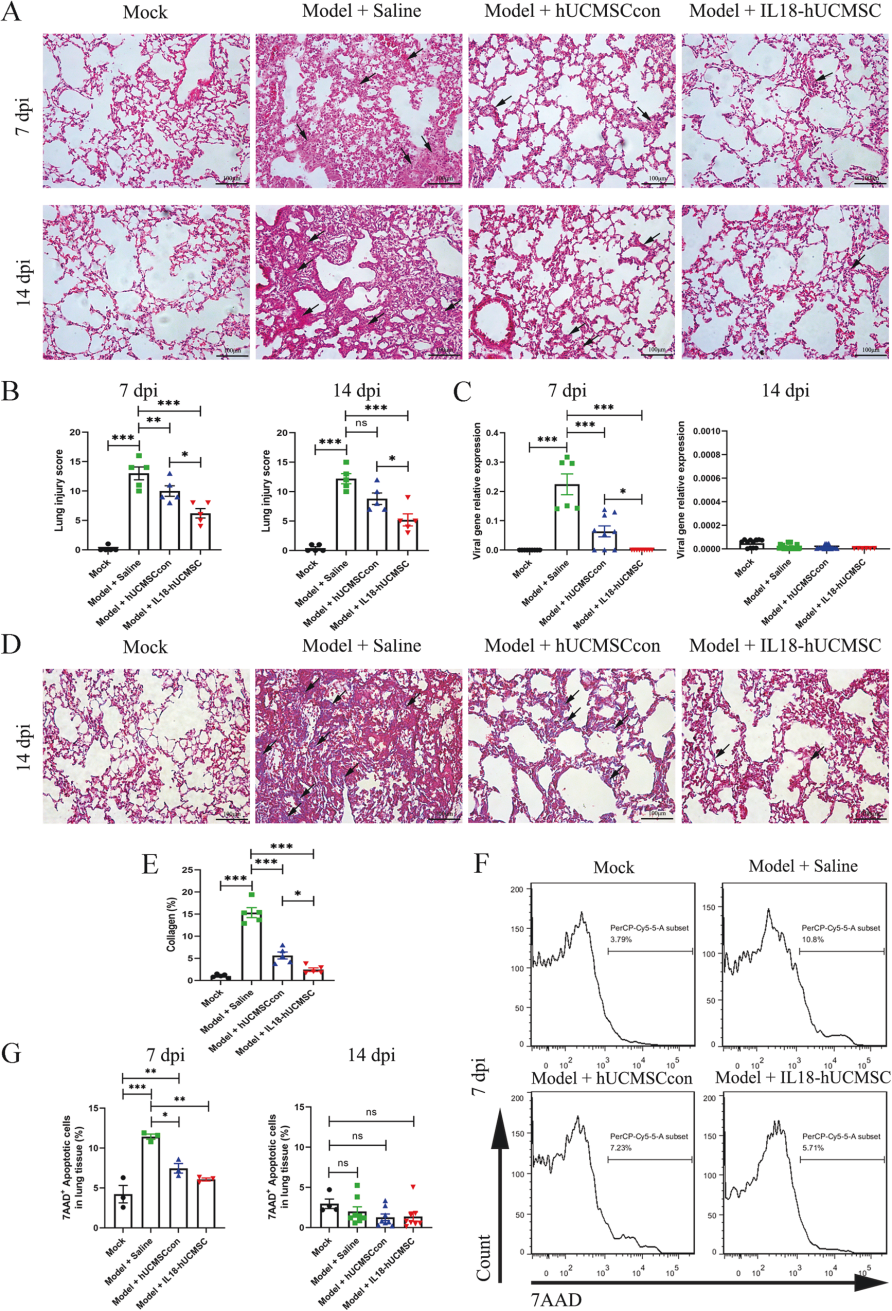
IL18-hUCMSC significantly ameliorated lung injury, fibrosis, and cell apoptosis at the cellular level. **A**, **B** Mice lung tissue was harvested at 7 dpi and 14 dpi, and lung sections were stained with H&E (**A**). Scale bars, 100 μm. Quantification of lung injury in each group (lung injury score) was calculated by analyzing alveolar edema (0–4 score), inflammation (0–4 score), bleeding (0–4 score), and interstitial tissue (0–4 score) with a total 16 scores (**B**). **C** Mouse lungs were examined for changes in viral load by analyzing the M1 expression of viral gene at 7 dpi and 14 dpi. **D**, **E** Collagen deposition of lung sections was assessed by staining for Masson’s trichrome at 14 dpi (**D**). Scale bars, 100 μm. Collagen deposition was used as a surrogate of fibrosis and was reported as a percentage of the septal area (**E**). **F**, **G** The cell apoptosis in lung tissues was analyzed by flow cytometry, and 7AAD^+^ cells were regarded as apoptotic cells (**F**); the percentages of 7AAD^+^ cells in different groups were calculated at 7 dpi and 14 dpi (**G**). The arrows in A and D showed the major features and changes of IHC images in different groups. Data are shown as mean ± SEM. *n* = 3–10 in each group. *p < 0.05, **p < 0.01, ***p < 0.001.

Histological examinations of lung tissues by HE staining showed the occurrence of alveolar edema, inflammation, bleeding, and interstitial tissue. PR8 infection induced severe alveolar edema, large infiltration of inflammatory cells, slight bleeding, and hyperplasia of interstitial tissue in the Model + Saline group; and hUC-MSCs administration could suppress the occurrence of these symptoms at 7 dpi and 14 dpi. Compared with hUCMSCcon treatment in the Model + hUCMSCcon group, IL18-hUCMSC significantly attenuated these four aspects of acute lung injuries in the Model + IL18-hUCMSC group (Fig. 4A). The histopathological scores significantly decreased by IL18-hUCMSC treatment compared to hUCMSCcon treatment; the suppression rate ranged from 10.00 ± 1.79 to 6.20 ± 1.60 at 7 dpi and from 8.80 ± 1.94 to 5.20 ± 2.04 at 14 dpi, respectively (Fig. 4B). To assess whether hUC-MSCs regulate viral replication in damaged lungs, qPCR was used to detect changes in the viral matrix protein 1 (M1) expression in the lungs of PR8-infected mice, which could indirectly reflect the viral load. The viral load in the lungs of the Model + Saline group greatly increased after PR8 infection at 7 dpi, and hUC-MSCs treatment significantly reduced M1 expression. The M1 gene was barely expressed in the Model + IL18-hUCMSC group, which demonstrated that IL18-hUCMSCs have an antiviral function (Fig. 4C). In addition, we could not find M1 expression in any group at 14 dpi (Fig. 4C). Moreover, collagen deposition was analyzed in the lung tissue at 14 dpi using Masson’s Trichrome staining; we found that PR8 infection induced much lung fibrosis in the Model + Saline group, as indicated by the blue area in the pulmonary interstitium (Fig. 4D). hUC-MSCs injection significantly reduced fibrosis, in particular, IL18-hUCMSC showed enhanced performance. The percentages of collagen area were 15.34 ± 2.24%, 5.64 ± 1.56%, and 2.44 ± 0.80% in the Model + Saline group, Model + hUCMSCcon group, and Model + IL18-hUCMSC group, respectively (Fig. 4E). Figure 3F shows lung necrosis after PR8 infection. We next analyzed the cell apoptosis in lung tissue at 7 dpi and 14 dpi using 7AAD staining. The flow cytometric analysis was used to identify the percentage of 7AAD positive cells in all groups (Fig. 4F) and demonstrated that hUC-MSCs injection significantly decreased cell apoptosis at 7 dpi compared to the Saline treatment, but there was no obvious difference between hUCMSCcon and IL18-hUCMSC at 7 dpi and 14 dpi (Fig. 4G). Collectively, IL18-hUCMSC protected the lungs by reducing inflammation, fibrosis, and cell apoptosis at the cellular level.

### IL18-hUCMSC had better immunosuppression on T-cells in BALF

**Fig. 5:**
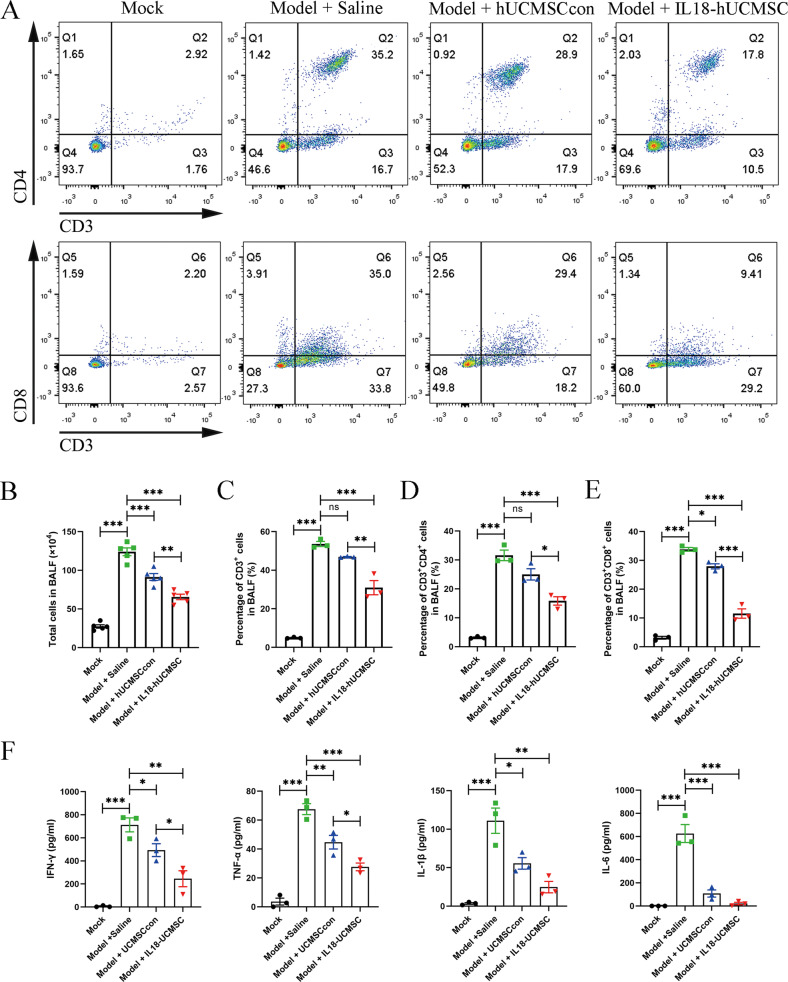
IL18-hUCMSC has a superior immunosuppressive effect on T-cells and the secretion of proinflammatory cytokines in BALF. **A** The CD3^+^ T-cell and its CD4^+^ and CD8^+^ subpopulations in BALF at 7 dpi were analyzed by flow cytometry. **B**–**E** The number of total cells in BALF was counted (**B**), and the percentage of CD3^+^ T-cell (**C**), CD4^+^ T-cell (**D**) and CD8^+^ T-cell (**E**) in BALF were calculated according to flow cytometric analysis. **F** The protein level of pro-inflammatory cytokines (IFN-γ, TNF-α, IL-1β, and IL-6) in BALF at 7 dpi was analyzed by ELISA kit. Data are shown as mean ± SEM. *n* = 3-5 in each group. *p < 0.05, **p < 0.01, ***p < 0.001, ns = not significant.

Next, the change of T-cells and their subpopulations in BALF were analyzed by flow cytometry after PR8 infection and hUC-MSCs treatment. The percentages of CD3^+^, CD4^+^, and CD8^+^ T-cells in BALF at 7 dpi were shown (Fig. 5A). Compared with Saline treatment in the Model + Saline group, IL18-hUCMSC largely reduced the number of total cells in BALF (Fig. 5B); further, we found that IL18-hUCMSC treatment could significantly decrease the infiltration of CD3^+^, CD4^+^, and CD8^+^ T-cells into BALF (Fig. 5C–E). In contrast, hUCMSCcon therapy effectively reduced CD3^+^ and CD4^+^ T-cells in BALF at 7 dpi (Fig. 5C, D). Meanwhile, the protein levels of four proinflammatory cytokines were evaluated in BALF at 7 dpi. The results demonstrated that IL18-hUCMSC treatment largely suppressed IFN-γ, TNF-α, IL-1β, and IL-6 expression in the Model + IL18-hUCMSC group compared with the Model + Saline group; but there was no statistical difference in IL-1β and IL-6 expression between hUCMSCcon and IL18-hUCMSC (Fig. 5F). Altogether, IL18-hUCMSC was a more effective immunosuppressant in BALF.

### IL18-hUCMSC has no enhanced performance in suppressing proinflammatory cytokine expression in serum and lung tissue

**Fig. 6:**
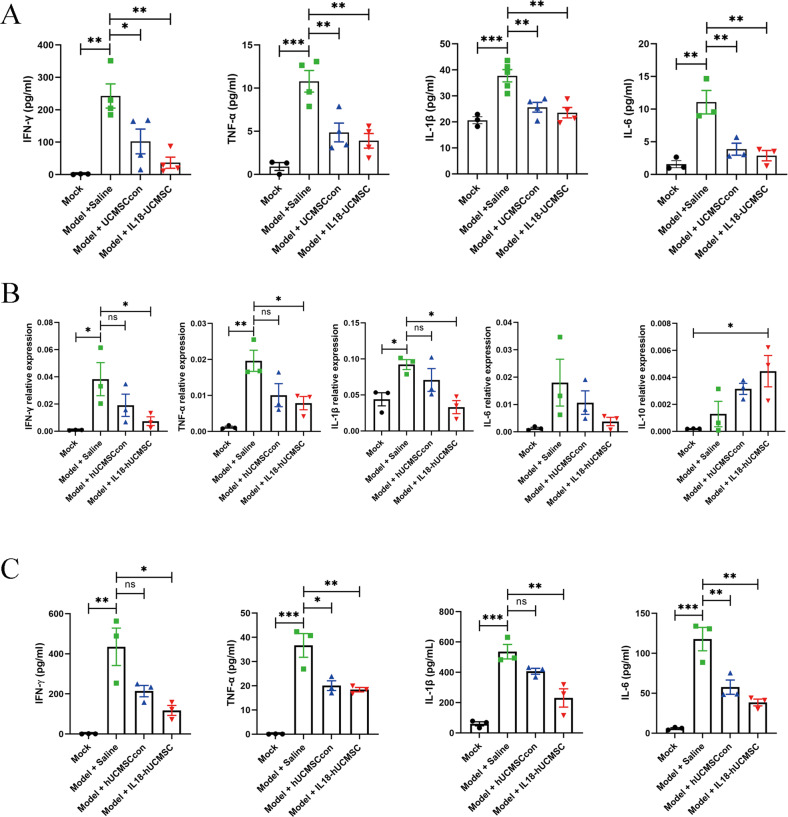
IL18-hUCMSC did not have a better immunosuppressive effect on the expression of proinflammatory cytokines in serum and lung tissue. **A** The protein level of pro-inflammatory cytokines (IFN-γ, TNF-α, IL-1β, and IL-6) in serum in different groups at 7 dpi were analyzed by ELISA kit. **B** The mRNA expression of IFN-γ, TNF-α, IL-1β, IL-6, and IL-10 in lung tissue was analyzed by qPCR. **C** The protein level of IFN-γ, TNF-α, IL-1β, and IL-6 in lung tissues in different groups at 7 dpi was analyzed by ELISA kit. Data are shown as mean ± SEM. *n* = 3–5 in each group. *p < 0.05, **p < 0.01, ***p < 0.001, ns = not significant.

PR8 infections in the lung typically induce systemic inflammation, while proinflammatory cytokines are also overexpressed in serum. Our results showed that hUC-MSCs therapy could significantly reduce proinflammatory cytokine expression in serum at 7 dpi compared with Saline treatment, but no difference was observed between hUCMSCcon and IL18-hUCMSC (Fig. 6A). Then, we assessed proinflammatory cytokine expression in the lung tissue. Compared to Saline treatment in the Model + Saline group, IL18-hUCMSC could significantly reduce mRNA expression of proinflammatory cytokines, especially IFN-γ, TNF-α, and IL-1β; meanwhile, IL-10 (anti-inflammatory cytokine) was more highly expressed in the Model + IL18-hUCMSC group (Fig. 6B). However, there was no obvious statistical difference between hUCMSCcon and IL18-hUCMSC (Fig. 6B). We also observed similar trends in the protein levels of the above proinflammatory cytokines in lung tissue homogenate (Fig. 6C). While IL18-hUCMSC treatment did not enhance performance in serum and lung tissue compared with hUCMSCcon, IL18-hUCMSC still had a therapeutic effect on N1N1 virus-induced lung damage.

### TGF-β1 participates the immunosuppression and anti-apoptosis pathways of IL18-hUCMSC

**Fig. 7:**
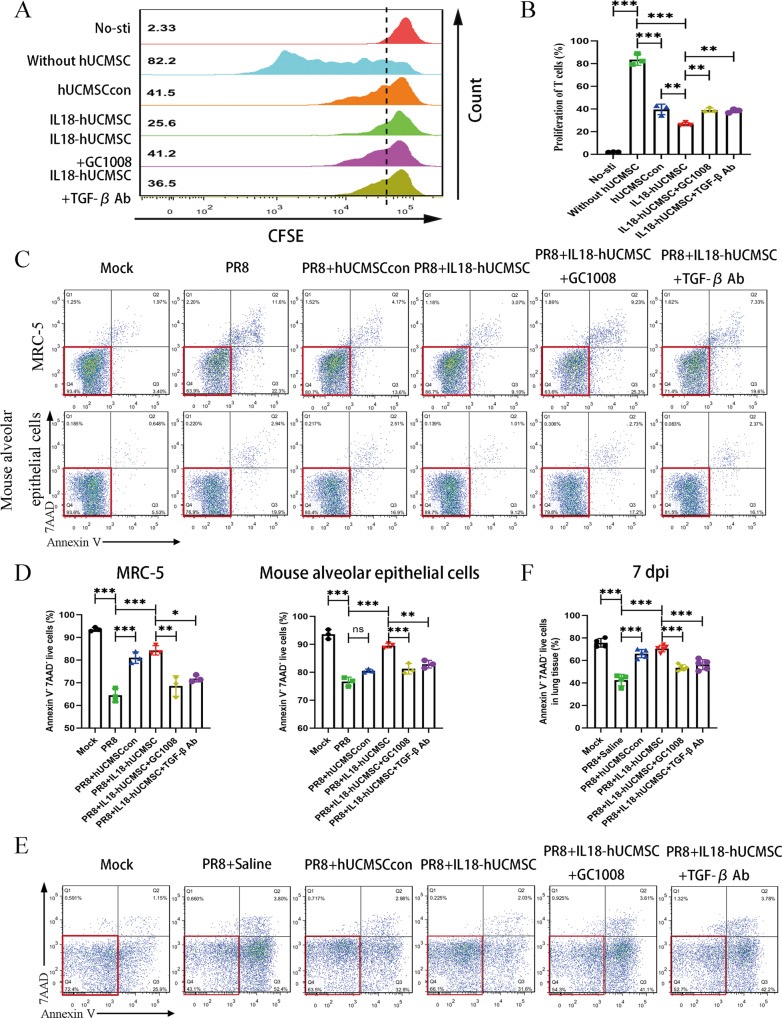
TGF-β1 is the key factor that participates the immunosuppression and anti-apoptosis pathways of IL18-hUCMSC in vitro and in vivo. **A**, **B** The proliferation level of human CD3^+^ T-cells was analyzed by flow cytometry; the change of CFSE fluorescence intensity indicates the growth ratio (A); and the immunosuppression ratio was analyzed statistically (**B**). **C**, **D** Representative flow cytometric images with Annexin V/7-AAD double staining assay of cells treated with or without PR8 virus (**C**); Quantification of Annexin V^-^7AAD^-^ live cells is shown: (Annexin V^-^7AAD^-^ cell amount / total cell amount) × 100% (**D**). Human MRC-5 cell line and mouse alveolar epithelial cells were used in apoptosis experiments in vitro. **E**, **F** The cell apoptosis in lung tissues was analyzed by flow cytometry, and 7AAD^+^ cells were regarded as apoptotic cells (**E**); the percentages of Annexin V^-^7AAD^-^ live cells in different groups were calculated at 7 dpi (**G**). Fresolimumab (GC1008) and TGF-β Ab is the blocking reagent that target to TGF beta-1,2,3. Data are shown as mean ± SEM. *n* = 3–5 in each group. *p < 0.05, **p < 0.01, ***p < 0.001, ns = not significant.

Considering TGF-β1 expression in IL18-hUCMSC group was significantly increased than that in hUCMSCcon and IFN-γ-hUCMSC (Supplementary fig. 4C), we next used Fresolimumab (GC1008) and TGF beta-1,2,3 monoclonal antibody to investigate the role of IL18-hUCMSC-derived TGF-β1 on immunosuppression and in reducing apoptosis. In the immunosuppression experiment, the blockade of TGF-β1-mediated immunosuppressive pathway with GC1008 and TGF-β Ab could obviously restore the proliferation of CD3^+^ T cells, when compared with IL18-hUCMSC treatment group (Fig. 7A, B). In the following anti-apoptosis experiments, we found that PR8 virus infection induce apoptosis of MRC-5 cell line and mouse alveolar epithelial cells in vitro, the percentage of Annexin V^-^7AAD^-^ live cells significantly decreased, compared with Mock group. IL18-hUCMSC and hUCMSCcon co-culture could largely increase the percentage of live MRC-5 cells, and only IL18-hUCMSC enhanced the percentage of live mouse alveolar epithelial cells. It demonstrated that IL18-hUCMSC has better cyto-protection function than hUCMSCcon. In addition, GC1008 and TGF-β Ab can inhibit this cyto-protection ability of IL18-hUCMSC, which is shown by a significant reduction of viable cells of MRC-5 and mouse alveolar epithelial cells (Fig. 7C, D). In vivo, the cell protection mechanism of IL18-hUCMSC-derived TGF-β1 was investigated in the mouse model of severe pneumonia. The results indicated that IL18-hUCMSC could indeed increase the number of living cells in lung tissues, and the blockade of TGF-β1 with GC1008 and TGF-β Ab largely reduced the ratio of living cells in lung tissues (Figs. 7E, F). Taken together, it demonstrated that TGF-β1 should be the key factor that participates the immunosuppression and anti-apoptosis pathways of IL18-hUCMSC in vitro and in vivo.

## Discussion

COVID-19 patients have increased levels of IL-18, which is involved in the generation of cytokine storms after SARS-CoV-2 infection [44]. Rodrigues TS et al. studied moderate and severe COVID-19 patients and found that inflammasome-derived products such as caspase-1 and IL-18 in the sera are correlated with markers of COVID-19 severity, including IL-6 and lactate dehydrogenase (LDH). Moreover, a higher level of IL-18 is associated with disease severity and poor clinical outcomes [45]. In this study, the mouse-adapted H1N1 influenza virus (A/Puerto Rico/8/34) was used to mimic SARS-CoV-2-induced pneumonia and lung injuries in mice. We also found higher levels of IL-18 than other cytokines, such as IFN-γ, TNF-α, IL-1β, IL-6, and IL-10, in a mouse model (Fig. 1C, D). In addition, the most severe form of acute lung injury is represented by acute respiratory distress syndrome (ARDS), which is commonly observed in severe COVID-19 patients [46]. Elevated IL-18 concentrations have been found in the serum and lungs of patients with ARDS (to the order of 600 pg/mL) and are correlated with severity score and death [47]. The protein levels of IL-18 in our mouse model almost exceeded 2000 pg/mL from 1 to 14 days after H1N1 infection (Fig. 1D). Together, they demonstrated that IL-18 could be important in cases of H1N1-induced severe pneumonia.

The IL‐18 precursor (pro-IL-18) is constitutively expressed within the cytoplasm of monocytes, macrophages, and dendritic cells, as well as in endothelial cells, keratinocytes, and intestinal epithelial cells of the gastrointestinal tract [48]. It is synthesized as an inactive precursor, processed to its active form by caspase‐1, and finally released [49]. Like IFN-γ, IL-18 also promotes the pro-inflammation process after binding to its receptor (IL-18R) and mediates the formation of the inflammatory microenvironment. Therefore, IL-18 was initially described as an interferon (IFN)γ-inducing factor. The inflammatory microenvironment is a prerequisite for MSCs to play an immunomodulatory role in vivo [50]. For example, the resting MSCs do not express indoleamine 2,3-dioxygenase (IDO), but they overexpress IDO to exert an immunosuppressive effect after IFN-γ activation. The ability of MSCs to adopt a different phenotype in response to special inflammatory microenvironments is crucial for understanding their potential for precise therapeutic treatment in immune-mediated disorders. Bernardo ME et al. found that MSCs can sense inflammation and adopt a proinflammatory or anti-inflammatory phenotype by interfering with innate and adaptive immune responses both in vitro and in vivo [15]. Therefore, understanding the specific inflammatory microenvironment of severe COVID-19 disease helps prime the appropriate cytokines to enhance the immunomodulatory potential of MSCs. In this study, IL-18 could be the superior candidate to prime hUC-MSCs to enhance the therapeutic efficacy of severe H1N1-induced pneumonia in mice.Many studies have found that cytokines that emerge in inflammatory microenvironments are typically used to prime MSCs to enhance specific properties, including IFN-γ, TNF-α, IL-1β, IL-17A, and IL-25 [13, 16–26]. Kim et al. reported that IFN-γ-primed MSCs are correlated with the induction of IDO expression in MSCs via the IFN-γ-JAK-STAT1 pathway, which suppresses T-cell proliferation during GvHD treatment [23]. Bai et al. found that IL-17A pretreatment enhances the efficacy of MSCs on mice with ischemia-reperfusion acute kidney injury (IRI-AKI) by increasing the Treg percentages through the COX-2/PGE2 pathway [25]. Importantly, our results found that IL-18-primed hUC-MSCs enhance immunosuppression ability on the proliferation of T-cells and their subpopulations in vitro and in vivo, partly via the TGF-β1-mediated regulatory pathway (Fig. 2E–G). However, IDO, PGE2, and TSG-6 expressions did not change (Supplementary Fig. 3C). Regarding trophic factors, we tested five growth factors, including NGF, IGF-1, EGF, FGF-2, and HGF. Only NGF expression increased after IL-18 priming, but IGF-1, EGF, and FGF-2 had no statistical changes (Supplementary Fig. 3D), which means that IL-18 priming does not significantly influence most trophic factors. While Redondo-Castro et al. reported that IL-1α and IL-1β (which belongs to the IL-1 family, like IL-18) did not affect VEGF, NGF, BDNF, or IL-1Ra expression but induced strong G-CSF release from MSCs [18]. In addition, we first reported that IL-18 priming could enhance the proliferation of hUC-MSCs, but not migration in a scratch wound assay (Fig. 2B–D). IL-18-primed hUC-MSCs in our study have special characteristics, including enhanced proliferation and immunosuppressive ability and increased expressions of TGF-β1, NGF, MMP-1, VCAM-1, and many chemokines.

We believe that priming is needed in vitro, even though IL-18 was found in vivo in a huge amount. Multiple pro-inflammatory cytokines have been detected to have a certain amount of expression in the pneumonia model, such as TNF-α, IFN-γ, IL-1β, and IL-6. MSCs could be activated or primed by these factors alone or in combination, and different factors have different biological effects on MSCs. Although IL-18 was found in mice in huge amounts, MSCs can also be affected by other low expression factors at the same time. As a result, it is difficult for us to control the biological function of MSCs in vivo. In addition, similarly to immune cells [51], MSCs have been shown to ‘remember’ a stimulus after transitioning to new environments [15]. Therefore, MSCs have been primed to trigger a ‘short-term-memory’ effect (mimicking microenvironmental stimuli) in vitro, thus avoiding the need for in vivo activation of the MSCs when aiming toward specific therapeutic activities. That’s why we need to prime MSCs with a specific factor (like IL-18) in vitro, and explore the unique therapeutic mechanism of IL-18-primed MSCs in vivo.

In this study, we reported that IL18-hUCMSCs could significantly ameliorate some symptoms of pneumonia, such as weight loss, death, lung injury, lung fibrosis, and apoptosis of lung cells. Considering the therapeutic mechanism of IL-18-primed hUC-MSCs, this is likely due to the suppression of the inflammatory response. We found that IL18-hUCMSCs have better immunosuppressive effects on T-cell infiltration (CD3^+^, CD4^+^, and CD8^+^ T-cells) and inflammatory cytokine secretion (IFN-γ and TNF-α) in BALF (Fig. 5). However, IL18-hUCMSCs did not show superior inhibition of inflammatory cytokine expression in serum and lung tissue compared to hUCMSCcon therapy; IL18-hUCMSCs still have better immunosuppressive performance than the Saline treatment group (Fig. 6). Considering the molecular mechanism of IL18-hUCMSCs in its superior effects, we found that TGF-β1 significantly increased after IL-18 priming in the IL18-hUCMSC group than hUCMSCcon and IFN-γ-hUCMSC group; but IDO and PD-L1 are the key immunosuppressive factors in IFN-γ-hUCMSC (Supplementary Fig. 4C). As reported, TGF-β1 is an essential regulator of cell proliferation, survival, and apoptosis; and TGF-β1 has anti-apoptotic effects in myelo-monocytic leukaemic cells co-cultured with stromal cells [52]. In addition, in an LPS-induced ARDS mouse model, MSCs overexpressing TGF-β1 could regulate lung inflammation and attenuate lung injuries by modulating the imbalance of Th17/Treg in the lungs [53]. We also found TGF-β1 participate the immunosuppression and anti-apoptosis pathways of IL18-hUCMSC in vitro and in vivo, by using the Fresolimumab (GC1008) and TGF beta-1,2,3 monoclonal antibody to block the activity of TGF-β1 (Fig. 7).

In conclusion, IL-18 is highly expressed in H1N1-induced severe lung injury in mice, and it is an appropriate cytokine to prime hUC-MSCs in vitro to improve precision therapy against viral-induced pneumonia, such as COVID-19.

## Supplementary information

Supplementary materials

Reproducibility checklist

## Data Availability

All reagents used in this work are available upon request and a brief statement describing the purpose of their use. Data in this study are available upon request from the corresponding author.
