# Supplementary material for: Interleukin-18-primed human umbilical cord-mesenchymal stem cells achieve superior therapeutic efficacy for severe viral pneumonia via enhancing T-cell immunosuppression
Source: Cell Death Dis. 2023 Jan 28;14(1):66. doi: 10.1038/s41419-023-05597-3 (PMC9883134; doi:10.1038/s41419-023-05597-3)
Supplement: Supplementary file 1 — Supplementary materials [file 41419_2023_5597_MOESM1_ESM.pdf]

Supplementary materials

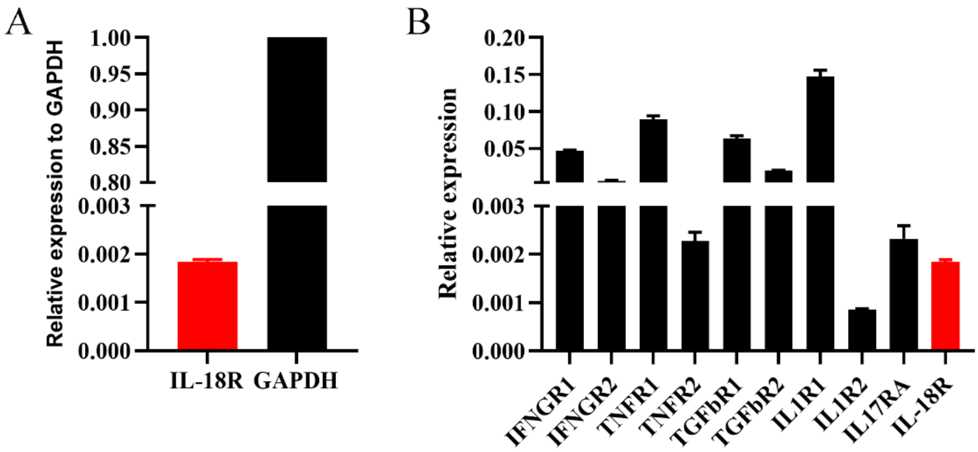

Supplementary Figure 1. The IL-18 receptor (IL-18R) was constitutively expressed in hUC-MSCs. (A) The relative mRNA expression of IL-18R was analyzed by qPCR, when GAPDH expression was regarded as 1. **(B)** The mRNA expression of several receptor of cytokines, including IFN-γ receptors (IFNGR1 and IFNGR2), TNF receptors (TNFR1 and TNFR2), TGF-β receptors (TGFbR1 and TGFbR2), IL-1 receptors (IL1R1 and IL1R2), IL-17 receptor (IL17RA), and IL-18 receptor (IL-18R). Data are shown as mean ± SEM. n = 3 in each group.

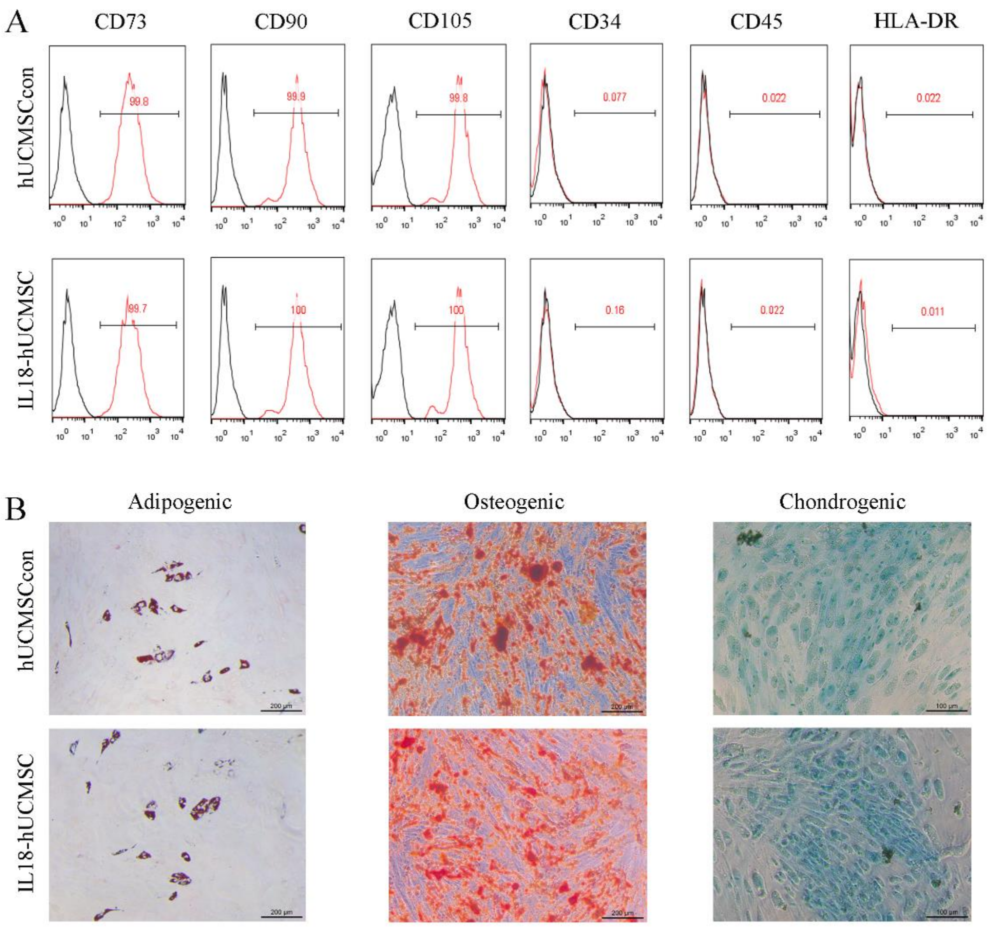

Supplementary Figure 2. IL-18 priming has no influence on surface marker and tri-lineage differentiation of hUC-MSCs. (A) The expressions of cell surface makers on P4 hUCMSCcon and IL18-hUCMSC were detected by flow cytometry. **(B)** Representative stained images showed the adipogenic, osteogenic, and chondrogenic potentials of P4 hUCMSCcon and IL18-hUCMSC, as confirmed by Oil red O, Alizarin red, and toluidine blue, respectively. Scale bars, 200 or 100 µm. n = 3 in each group.

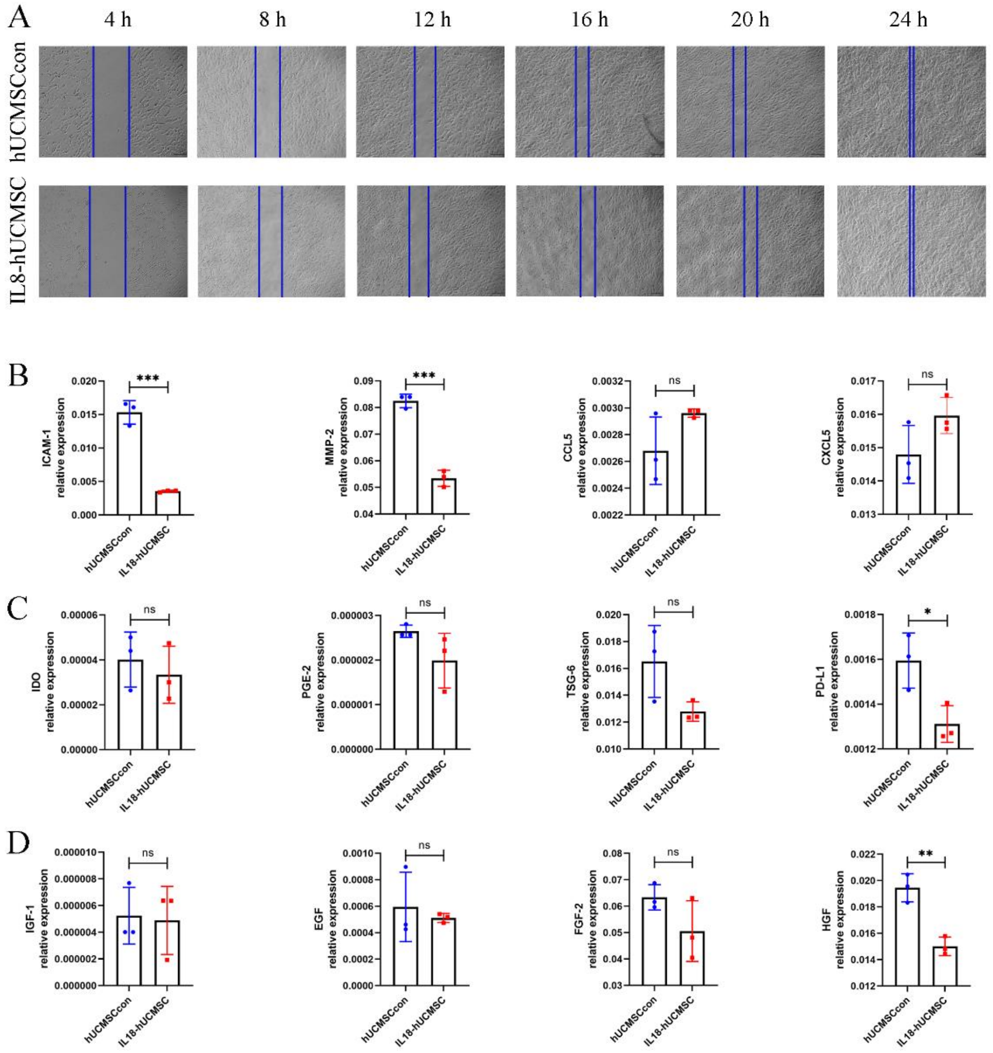

Supplementary Figure 3. The effects of IL-18 priming on migration and paracrine secretion of hUC-MSCs. (A) Representative images of the wound closure of hUC-MSCs in different groups by cell scratch assay at 4 h, 8 h, 12 h, 16 h, 20 h and 24 h. Scale bars, 200 μm. **(B-D)** The mRNA expression of immunomodulatory factors (IDO, PGE-2, TSG-6 and PD-L1) (B), adhesion factors and chemokines (ICAM-1, MMP-2, CCL5 and CXCL5) (C), and growth factors (IGF-1, EGF, FGF-2 and HGF) (D) of hUCMSCcon and IL18-hUCMSC were analyzed by qPCR. Data are shown as mean ± SEM. n = 3 in each group. $^{*}p < 0.05$, $^{**}p < 0.01$, $^{***}p < 0.001$, ns = not significant.

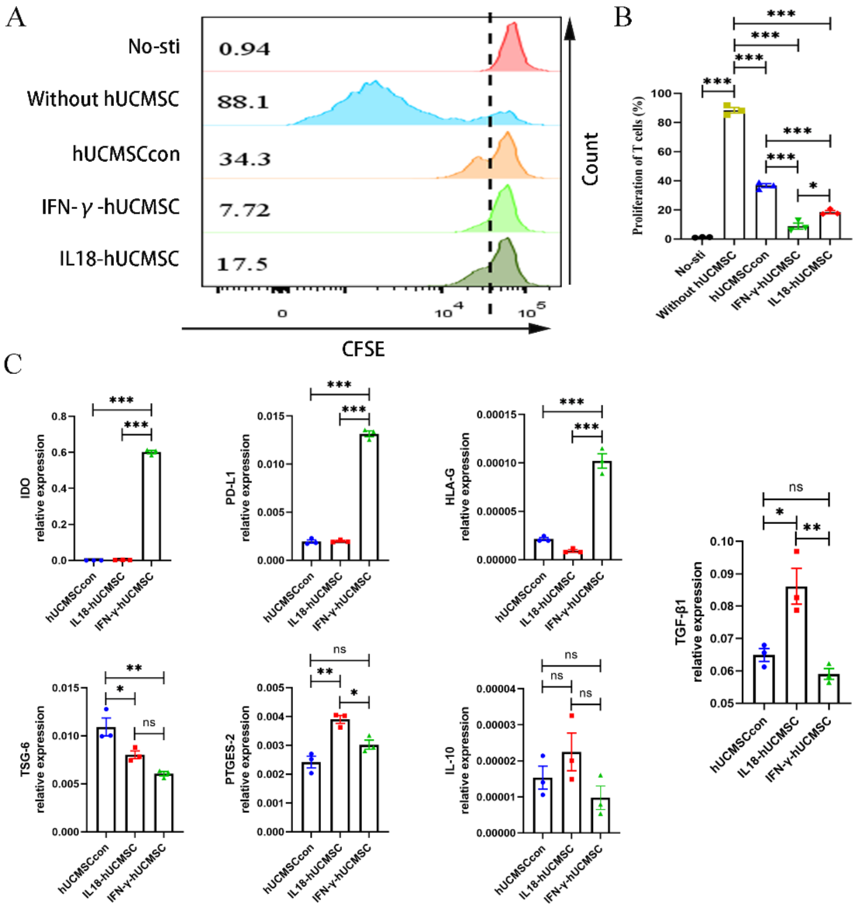

Supplementary Figure 4. The immunosuppressive potential and immunomodulatory genes expression were compared between IFN-γ-hUCMSC and IL18-hUCMSC. (A and B) The proliferation level of human $CD3^+$ T-cells was analyzed by flow cytometry; the change of CFSE fluorescence intensity indicates the growth ratio (A); and the immunosuppression ratio was analyzed statistically (B). **(C)** The mRNA expressions of immunomodulation-related genes (IDO, PD-L1, HLA-G, TSG-6, PTGES-2, IL-10, and TGF-β1) in hUCMSCcon, IL18-hUCMSC and IFN-γ-hUCMSC were analyzed by qPCR. Data are shown as mean ± SEM. n = 3 in each group. *$p < 0.05$, **$p < 0.01$, ***$p < 0.001$, ns = not significant.
